# Adult Stature Estimation from Radiographic Metatarsal Length in a Contemporary Korean Population

**DOI:** 10.3390/ijerph181910363

**Published:** 2021-10-01

**Authors:** Suyeon Park, Young Yi, Battur Tsengel, Jahyung Kim, Dong-Il Chun, Sung-Hun Won, Tae-Hong Min, Jeong-Hyun Park, Mijeong Lee, Jaeho Cho

**Affiliations:** 1 Department of Biostatistics, Soonchunhyang University Seoul Hospital, Seoul 04401, Korea; suyeon1002@schmc.ac.kr; 2 Department of Applied Statistics, Chung-Ang University, Seoul 06974, Korea; 3 Department of Orthopaedic Surgery, Inje University Seoul Paik Hospital, Seoul 04551, Korea; 20vvin@naver.com; 4 Institute for Skeletal Aging and Orthopedic Surgery, Chuncheon Sacred Heart Hospital, Hallym University, Chuncheon 24253, Korea; batturtkh@gmail.com; 5 Department of Orthopaedic Surgery, Armed Force Gangneung Hospital, Gangneung 25422, Korea; hpsyndrome@naver.com; 6 Department of Orthopaedic Surgery, Soonchunhyang University Seoul Hospital, Seoul 04401, Korea; orthochun@gmail.com (D.-I.C.); orthowon@gmail.com (S.-H.W.); minth916@gmail.com (T.-H.M.); 7 Department of Anatomy & Cell Biology, Graduate School of Medicine, Kangwon National University, Kangwon 24341, Korea; jhpark@kangwon.ac.kr (J.-H.P.); toff337@hanmail.net (M.L.); 8 Department of Orthopaedic Surgery, Chuncheon Sacred Heart Hospital, Hallym University, Chuncheon 24253, Korea

**Keywords:** anatomy, forensic sciences, forensic anthropology, identification, body height, metatarsal bones, linear model

## Abstract

The ability to estimate stature can be important in the identification of skeletal remains. This study aims to develop a Korean-specific equation predicting stature using radiographic measurements in the contemporary Korean population. 200 healthy Korean adults, including 102 males and 98 females, were randomly selected (age, range 20–86 years). The first and second metatarsals of the foot were measured by a standing X-ray using a digital medical image viewer. The result showed a statistically significant correlation between metatarsal length and stature in Korean populations (male, R = 0.46, *p* < 0.001; female, R = 0.454, *p* < 0.001). Values of correlation coefficients (R) of the equations were 0.431 to 0.477. Compared to equations derived from other races, the Korean-specific equation showed significantly lower error values for estimating the actual height of Koreans through cross-validation. In conclusion, this study is the first to propose a Korean-specific regression formula for estimating stature using metatarsal length and a verified formula for precise application to the Korean population. However, given the relatively low correlation coefficient, the stature estimation formula derived from this study can be utilized when other bones that allow more accurate stature estimation are not available.

## 1. Introduction

Recently, as the number of deaths and disappearances due to natural or massive disasters increases, the importance of forensic anthropological population data is also increasing. Although identification is one of the most important processes in dealing with disasters, the applicable items for identification are greatly limited when the human body is severely damaged or the skeleton is separated [1]. Sex, age, weight, and stature are important factors for personal identification of the remains of human skeletal [2,3,4,5]. In particular, the estimation of stature or living height has the main role in the analysis of unidentified human remains, since a person’s height is a unique biological profile [6].

The stature of skeletal remains has been commonly estimated either by anatomical methods or by mathematical methods. The anatomical method reconstructs statures including the soft tissue corrections with any specific assumption regarding body proportion. Although a software process of preparing a three-dimensional model by taking more photos of a specimen has been recently introduced, [7] this method has a limitation that skeletal remains must be preserved [8,9]. However, the mathematical method can estimate the stature with only one bone, because this method is based on a correlation between stature and certain bone dimensions [9,10]. In fact, given the proper equations, the mathematical method is considered more convenient compared to the anatomical method as it allows the immediate calculation of the stature even with an incomplete skeleton. [9]

The science of estimating stature from bones has been known continuously since the 19th century, and researchers around the world have been developing individual regressions for different populations [11]. Consequently, this is based on a positive linear relationship between stature and the length of various parts of the body, including long bones, metacarpals, metatarsals, vertebrae, pelvis, scapula, calcaneus, talus, and skull [11,12,13,14,15,16,17]. Although various bones have been used to estimate stature, the individual long bones of the upper (humerus, ulna, radial) and especially lower (femur, tibia, and fibula) limbs are most widely used for derivation of regression formulas for stature estimation with accurate results [18,19,20,21]. However, there were reports of limitations in measuring long bones in forensics, as they are more likely to be found brittle and fragmented, making accurate measurements sometimes impossible. As small, long, or short bones among skeletal remains are more likely to be preserved by shoes or clothing along with their tissue characteristics, the usefulness of a method for estimating adult stature from foot bones has been suggested [22,23,24].

For the reasons discussed above, studies for stature estimation using foot bones, particularly metatarsals, have been published for different populations and regions [25,26,27,28]. The formula developed by Byers [25] using cadavers has been routinely used in forensics for over 20 years, but it is clear that it is a reference population that provides some degree of specificity. Skeletal development is influenced by a number of factors, such that the ratios between the various bones vary not only by race but also by geographic area [29]. It is also well known that issues such as race or ethnicity directly affect the regression formula for estimating stature. Therefore, to increase accuracy, it is necessary to obtain an appropriate formula for each population area. Furthermore, this issue need has been demonstrated by the differences that arise when used in different populations [26].

For other races, there have already been reports of the correlation between the metatarsal length and stature and the development of a formula for estimating the stature using the metatarsal. However, despite the suggestion that it is appropriate to use Korean-specific equations for the stature estimation of the Korean population, to our best of knowledge, there is no study using metatarsal length in the Korean population [30].

This study aims to confirm the correlation between the metatarsal length and stature in the Korean population and to develop a Korean-specific equation estimating stature using metatarsals. A prediction formula was constructed based on radiographic measurements of the maximum length of the metatarsal bones in this study.

## 2. Material and Methods

### 2.1. Subjects

The ethical approval was obtained from the Institutional Ethics Committee (CHUNCHEON 2021-01-010-001). Consent from all subjects was obtained prior to the study. Assuming a significance level of 0.01, a power of 99% and a dropout rate of 50% [28], 200 samples are required to satisfy an R^2^ value of 0.322 to 0.416.

The present research was conducted in the orthopedics department and was included a total of 200 healthy Korean adult volunteers (102 males and 98 females). The volunteers were recruited from two cities (Seoul and Chuncheon) in Korea. All persons who could interfere with measurements of stature and metatarsal lengths, such as skeletal deformities, fractures, or pathologies, were excluded from this study. Their ages ranged between 20 and 86 years and all were of Korean descent. The lower age limit was 20 years to be sure of completion of skeletal development and attaining maximum growth and the maximum length of different body parts.

### 2.2. Measurement of Stature

**Figure 1:**
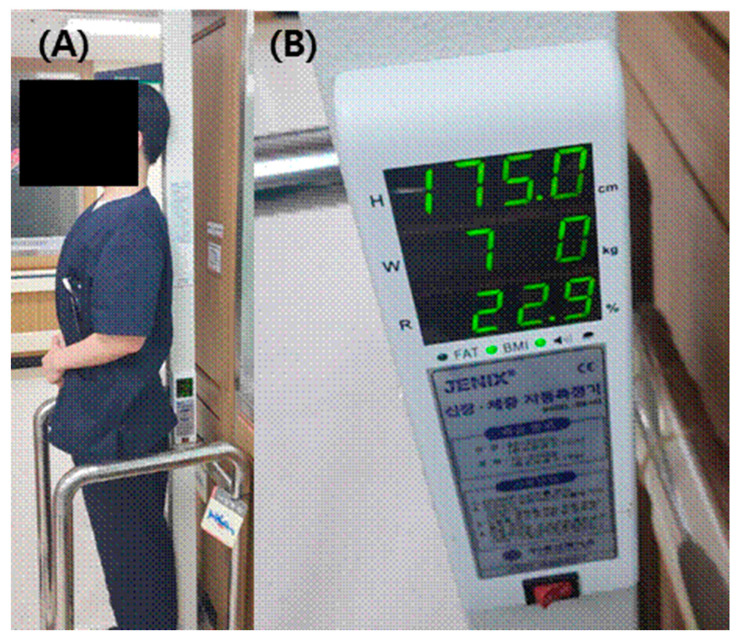
(**A**) Subjects stand upright in an anatomical position for measuring height. (**B**) The subject’s living height was measured by using the standard height measurement scale taken in centimeters (cm) (with one decimal place).

The subject’s living height was measured utilizing the standard height measurement automatic digital scale (DS-103, DongSahn Jenix, Seoul, Korea) taken in centimeters (cm) (with one decimal place). Subjects were required to stand upright in an anatomical position [11] (Figure 1). Stature was measured as the vertical distance from the vertex to the foot. Measurement was taken by making the subject stand erect barefoot in an upright posture on a horizontal resting plane while facing the front with his back against a scaling instrument. The back was extended and arms held to the sides. The instrument vertical plane came in contact with the participant’s head, buttocks, and heels.

### 2.3. Measurement of Metatarsal Bone

The first and second metatarsal lengths of the subject’s feet were measured with a standing dorso-plantar X-ray using the Picture Archiving and Communication System (PACS, Infinitt M6 version, Infinitt, Seoul, Korea), a digital medical image viewer commonly used in hospitals.

**Figure 2:**
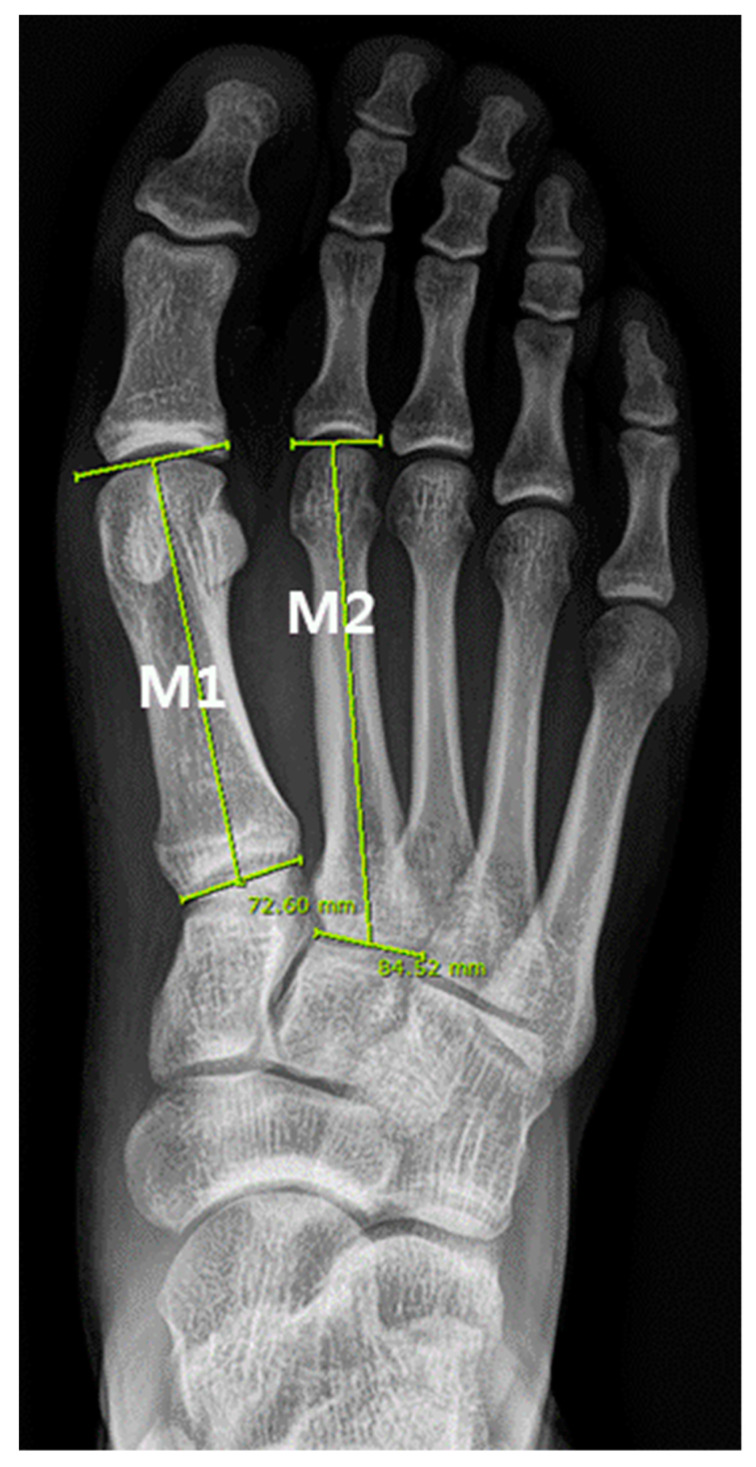
Digital measurements (green lines) were used to calculate metatarsal length: 1st and (M1) and 2nd (M2) metatarsal bone.

Digital measurements were calibrated by the PACS system itself, but to minimize errors during the measurement of metatarsal lengths using PACS, all measurements were performed by two researchers. The average value of measurements of two researchers was used, and each researcher measured twice within one week and used the average value of two times (Figure 2).

According to the previous studies, [26,27,28] the definition of metatarsal length measurement was as follows.

M1—Maximum length of 1st metatarsal—the distance between the tip of the tuberosity and the most distal point of the head.

M2—Maximum length of 2nd metatarsal—the distance between the proximal tip and the most distal point of the head.

### 2.4. Statistical Analysis

Inter- and intra-observer reliabilities for all measurements were calculated by the intraclass correlation coefficient (ICC). According to the definition of Landis and Koch [31], ICCs of 0.81 to 1.00, 0.61 to 0.80, 0.41 to 0.60, 0.21 to 0.40, and 0.00 to 0.20 were interpreted as excellent, good, moderate, fair, and poor, respectively. The data analysis included means (m), standard deviations (SD), the correlation coefficient (R), standard error of estimate (SEE), adjusted determination coefficient (adj R^2^), and Linear Regression Model (LRM). Spearman correlation analysis was performed to estimate the relationship between the actual value and each predicted value. Wilcoxon signed-rank test was performed to assess whether the difference between the actual and predicted values is zero. As our model results could be overestimated, we further evaluated the accuracy of our model using mean squared error (MSE) values and cross-validation methods. MSE was calculated in all formulas as a method of measuring the mean square difference between the estimated value and the actual value. That is, it was used to evaluate the accuracy of each formula. To identify the interval validation in the proposed formula, we also performed the leave-one-out cross-validation (LOOCV) where the number of folds equals the number of instances in the data set. Thus, the machine learning algorithm is applied once for each instance, using all other instances as a training set and using the selected instance as a single-item test set. A *p*-value < 0.05 was considered to indicate a significant difference. Data processing and statistical analyses were performed by R version 3.3.1 and Rex software (http://rexsoft.org/ accessed on 13 August 2021).

## 3. Result

Intraclass correlation coefficients were generated for all measurements. All measurements were higher than 0.8 (indicating acceptable reliability) and were employed in the study (Table 1).

Table 2 shows the descriptive analysis of male and female measurements in both groups. Men (*n* = 102) have an average age of 48 ± 17.95 years and women (*n* = 98) have an average age of 49.5 ± 16.15 years. The stature differed significantly for corresponding male–female values (*p* ≤ 0.05) by independent samples *t*-test. The average stature was found to be about 124.45 mm greater in males than females.

**Figure 3:**
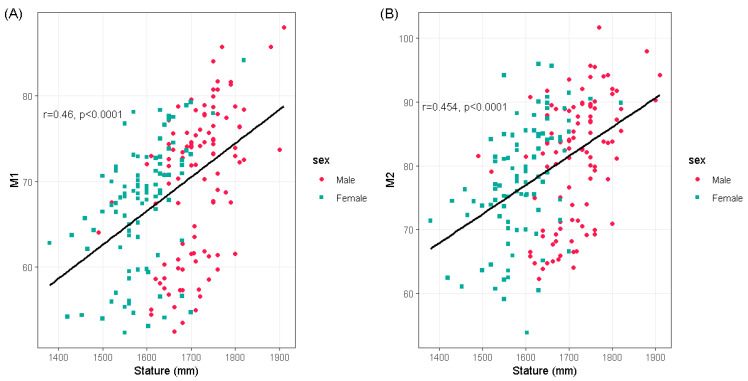
Relationship between the length of the metatarsal bones and stature in males and females, the relationship between the length of the first metatarsal and stature is shown in (**A**) and between the length of the second metatarsal and stature is shown in (**B**). There is a positive correlation between the length of both bones and stature (*p* < 0.0001).

Figure 3 shows the relationships between the stature and lengths of the first and second metatarsal lengths in the men and women, respectively. According to Table 3, Table 4 and Table 5, R was significant in all cases (*p* < 0.001). The stature was correlated with the size of the metatarsal bone in both genders. However, stronger correlations were detected between the stature and lengths of the metatarsal bone (M1; R = 0.4758, M2; R = 0.4773) in female. The highest correlation with stature was M2 for females (maximum length of 2nd metatarsal). The corresponding regression equation is as follows: S (Stature) = 1294.32 + 3.81M2, R = 0.4773.

## 4. Discussion

The first contribution of the present study is to propose a unique Korean regression equation for estimating height using metatarsal length after proving the correlation between metatarsal length and height for the first time in Koreans. The second is the unique data (including means, standard deviations, the correlation coefficient, standard error of estimate, adjusted determination coefficient, and linear regression model) of Korean populations for comparison with formulas derived from other races.

Stature estimation is one of the important factors of personal identification. The use of the proposed formula to predict the stature of a particular population is not suitable for other populations because regions have different gender, race, climate, and nutrition [32]. The equations of previous studies on stature estimation developed by Trotter and Gleser [21] and Trotter and Gleser [29] have been widely used worldwide by several researchers, and even in Korea until recently. However, at present, these regression formulas are no longer valid for representing different populations and generations. Their application can lead to inaccuracies in stature estimation in the current forensic field [33]. As the most accurate result of estimating height should be given by the population from which the equation was derived, each population should have its own regression formula using modern population samples to account for the historical trends in height [15]. Therefore, this study was conducted to estimate the stature of the contemporary Koreans living in the modern era.

In forensic or legal assessment, a mathematical method for estimating stature from skeletal remains is based on a positive linear relationship between stature and the length of various parts of the body [34]. In particular, the individual long bones of the lower (femur, tibia, and fibula) limbs have most commonly been used in the derivation of regression equations for estimating stature, and the regression formulas with high accuracy have been reported [18,19,20,21]. Lower limb measurements for the stature prediction were also more accurate than the foot measurement [35]. Although long bones of the lower limb are the most reliable for estimating stature, in practice, they are more likely to be fragmented in a way that prevents accurate assessment. On the other hand, it is important to develop a reliable method for determining adult stature from foot bones because small bones have a high probability of preservation. Several studies have been conducted on both the hand and foot bones, which are small bones, but the metatarsal has been suggested as the most appropriate [2,3,12,25,26,35]. With regard to the above-mentioned considerations, this study performed for the first time a study on radiographic metatarsal length to estimate the stature of the Korean population.

The formulas to estimate stature using radiographically determined metatarsal length have been reported in Spanish and Egyptian population groups [27,28]. Values of correlation coefficients (R) were lower in our study than those obtained from other published studies in different population groups (Spain and Egypt), and the regression formula was completely different. The formula for Spanish [28] had moderate relationships with correlation coefficients (R) that ranged between 0.567 and 0.783, whereas the formula for Egyptian had slightly higher values with correlation coefficients (R) ranging between 0.746 and 0.89. Our formula showed a correlation coefficient (R) ranging from 0.431 to 0.477, which is thought to indicate differences by race in the referenced population.

However, the results in our study cannot be interpreted that the formulas derived from our study are unavailable. Even in our formula, the correlation between metatarsal length and stature was statistically significant, but the correlation coefficient (R) was slightly lower than that of other races. Although studies on estimating the stature of the Korean population are very rare, it has been reported that the R-value in the formula for estimating the stature by measuring the femur length from cadavers using computed tomography was high, ranging from 0.85 to 0.89 [36]. Consequently, our results indicate that metatarsal length from digital radiographs do not provide a very highly reliable method for estimating stature in the Korean population. However, in cases in which the long bones of the extremities do not exist or analysis using long bones cannot be conducted, the regression formulas derived in this study may be used to estimate the stature.

To complete validation of the formula obtained for the Korean population, the actual value and each predicted value were compared at first. In the model that considered both male and female, when M1 was considered as a predictor, there was no significant difference in all models, and when M2 was considered as a predictor, there was a significant difference in Spanish_all_M2 (*p* < 0.001) and Egyptian_all_M2 (*p* < 0.001), respectively. The model considering only males showed the same results, and in the model considering only females, there was also a significant difference in Spanish_female_M1 (*p* = 0.0237). Second, to verify whether the formula proposed in this study is an objective and measurable model, intra- and inter-racial validation analyses were performed. To confirm the bias of our formula, the mean square error (MSE) and the cross-validation error (CVE) values were calculated using the actual height of Koreans. We also confirmed that the CVE (or MSE) of our formula was significantly lower than that of other formulas when estimating the actual height of Koreans using previously published formulas for estimating the heights of Spanish and Egyptians by the radiographically determined length of the metatarsals. The results were confirmed equally in both M1 and M2 in both sexes. Third, the leave-one-out cross-validation (LOOCV) was also performed to verify the estimated accuracy of the formula within the Korean population (Table 6). As can be inferred from the data shown, the formulas proposed in this study using measurements obtained in first and second metatarsal bones are significantly more accurate in estimating the stature of the Korean population compared to the formulas derived from other races. Therefore, this study is meaningful in that it provides Korean-specific regression formula for estimating stature using metatarsal length applicable to contemporary Koreans. That also means that regional studies are very much needed as racial and ethnic variations arise in different regions.

In stature estimation in a living person, many factors can influence this estimate. The age is one of them and must be at least 20 years old. All study subjects were adults over 20 years of age to ensure the fusion of the epiphyses [21], so the formula cannot be used for those under 20 years of age. Bodyweight was not considered as an influencing factor in this study [2]. In the literature review, a statistically significant correlation was observed between bodyweight and foot dimensions of both sides in both sexes [37] and the load-bearing capacity of the foot changed with respect to the weight of the body [38]. Thus, it is considered that further study considering body weight is needed in the future. Radiographic measurement of the bones of a living person offers the advantage of utilizing radiology in the display of skeletal structures without removing the surrounding soft tissue to avoid damage to the body. However, the method of indirectly measuring using radiographs may have limitations that may differ from the actual bone size because structures overlap in the radiographic image [39,40]. So, further study is needed to derive a formula for estimating the stature by measuring the actual metatarsal length in a cadaver with known antemortem stature.

## 5. Conclusions

First, a unique Korean regression equation for estimating height using metatarsal length was proposed. In addition, through validation analysis, the previous formula de-rived for other races proved to be inappropriate for the Korean population. Given the relatively low correlation coefficient, the stature estimation formula derived from this study can be utilized when other more accurate skeletal elements, such as intact long bones, are unavailable for analysis.

## Figures and Tables

**Table 1:** Inter- and intraobserver reliabilities for all measurements calculated by the intraclass correlation coefficient (ICC).

			ICC	95% CI.LB	95% CI.UB
Intra-observer	M1	Researcher 1	0.9866	0.9823	0.9898
		Researcher 2	0.9914	0.9879	0.9938
	M2	Researcher 1	0.9947	0.993	0.996
		Researcher 2	0.9927	0.9898	0.9948
Inter-observer	M1		0.9918	0.9885	0.9942
	M2		0.9942	0.9919	0.9959

Abbreviations: CI: confidence interval, M1: first metatarsal bone, M2: second metatarsal bone.

**Table 2:** Summary statistics (mean, standard deviation, median, and range) of age, stature, M1 (first metatarsal maximum length), and M2 (second metatarsal maximum length) measurements (mm) of both sexes.

	Female (*n* = 98)			Male (*n* = 102)		
	Mean ± SD	Median (IQR)	Range	Mean ± SD	Median (IQR)	Range
Age (years)	49.5 ± 16.15	52.5 (37.25, 61)	20 to 86	48 ± 17.95	51 (30.25, 61)	20 to 80
Stature (mm)	1590.51 ± 71.38	1580 (1550, 1630)	1380 to 1820	1714.96 ± 68.93	1710 (1670, 1750)	1490 to 1910
M1 (mm)	67.27 ± 7.21	68.59 (62.89, 71.99)	52.34 to 84.16	70.04 ± 8.57	72.57 (61.57, 75.95)	52.48 to 87.99
M2 (mm)	77.66 ± 8.93	77.62 ( 72.41, 84.4)	53.77 to 95.98	81.09 ± 9.61	82.71 (71.43, 88.93)	62.23 to 101.72

Abbreviations: IQR: Interquartile range, M1: first metatarsal bone, M2: second metatarsal bone, *n*: number, SD: standard deviation.

**Table 3:** Regression formulae for females with its correlation coefficient (R), adjusted determination coefficient (Adj R^2^), and standard error of estimate (SEE).

Formula	R	Adj R^2^	SSE
S = 1273.83 + 4.71M1	0.4758	0.2183	62.46033
S = 1294.32 + 3.81M2	0.4773	0.2198	62.40082

Abbreviations: S: Stature, M1: 1st metatarsal maximum length in mm, M2: 2nd metatarsal maximum length, in mm.

**Table 4:** Regression formulae for males with its correlation coefficient (R), adjusted determination coefficient (Adj R^2^), and standard error of estimate (SEE).

Formula	R	Adj R^2^	SSE
S = 1451.44 + 3.76M1	0.468	0.2112	60.61575
S = 1464.14 + 3.09M2	0.4312	0.1778	61.88643

Abbreviations: S: Stature, M1: 1st metatarsal maximum length in mm, M2: 2nd metatarsal maximum length, in mm.

**Table 5:** Regression formulae for unknown sex with its correlation coefficient (R), adjusted determination coefficient (Adj R^2^), and standard error of estimate (SEE).

Formula	R	Adj R^2^	SSE
S = 1285.76 + 5.36M1	0.4597	0.2074	83.02603
S = 1295.4 + 4.52M2	0.4538	0.2019	83.3114

Abbreviations: S: Stature, M1: 1st metatarsal maximum length in mm, M2: 2nd metatarsal maximum length, in mm.

**Table 6:** Mean square error (Korean versus Spanish and Egyptians) and cross-validation error (Korean validation for the total sample).

	Mean Square Error	Cross-Validation Error	Correlation Coefficient ^¥^	*p*-Value ^†^
Our_all_M1	6893.322	7033.284	0.4767	0.9475
Spanish_all_M1	9060.709		0.4767	0.379
Egyptian_all_M1	9336.686		0.4767	0.2299
Our_all_M2	6940.789	7081.715	0.4523	0.899
Spanish_all_M2	22,244.97		0.4523	<0.001
Egyptian_all_M2	20,915.28		0.4523	<0.001
Our_male_M1	3674.27	3822.71	0.4828	0.8951
Spanish_male_M1	9606.378		0.4828	0.8793
Egyptian_male_M1	8952.693		0.4828	0.6391
Our_male_M2	3829.931	3984.66	0.4601	0.8399
Spanish_male_M2	30,541.19		0.4601	<0.001
Egyptian_male_M2	26,820.46		0.4601	<0.001
Our_female_M1	3901.293	4065.54	0.5106	0.8496
Spanish_female_M1	4590.316		0.5106	0.0237
Egyptian_female_M1	5980.628		0.5106	0.0601
Our_female_M2	3893.863	4057.797	0.5152	0.9252
Spanish_female_M2	6878.444		0.5152	<0.001
Egyptian_female_M2	14,414.39		0.5152	<0.001

Abbreviations: M1: first metatarsal bone, M2: second metatarsal bone.; ^†^ *p*-value by Wilcoxon signed-rank test and the difference between the actual value and each predicted value was analyzed.; ^¥^ spearman correlation analysis was performed.

## Data Availability

The datasets used and/or analyzed during the current study are available from the corresponding author on reasonable request.
